# LaZn_12.37 (1)_, a zinc-deficient variant of the NaZn_13_ structure type

**DOI:** 10.1107/S1600536811028893

**Published:** 2011-07-30

**Authors:** Igor Oshchapovsky, Volodymyr Pavlyuk, Grygoriy Dmytriv, Fraser White

**Affiliations:** a Department of Inorganic Chemistry, Ivan Franko Lviv National University, Kyryla i Mefodia 6,79005 Lviv, Ukraine; b Agilent Technologies UK Ltd, 10 Mead Road, Oxford Industrial Park, Yarnton, Oxfordshire, OX5 1QU, England

## Abstract

The title compound (lanthanum dodecazinc), LaZn_12.37 (1)_, is confirmed to be a nonstoichiometric (zinc-deficient) modification of the NaZn_13_ structure type, in which one Zn atom (Wyckoff site 8*b*, site symmetry *m* 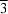) has a fractional site occupancy of 0.372 (11). The other Zn atom (96*i*, *m*) and the La atom (8*a*, 432) are fully occupied. The coordination polyhedra of the Zn atoms are distorted icosa­hedra, whereas the La atoms are surrounded by 24 Zn atoms, forming pseudo-Frank–Kasper polyhedra. Electronic structure calculations indicate that Zn—Zn bonding is much stronger than La—Zn bonding.

## Related literature

For general background to inter­metallics, see: Berche *et al.* (2009 ▶); Oshchapovsky *et al.* (2010 ▶); Pavlyuk *et al.* (2009 ▶); Rolla & Iandelli (1941 ▶). For isotypic structures, see: Iandelli & Palenzona (1967 ▶); Kuz’ma *et al.* (1966 ▶); Veleckis *et al.* (1967 ▶). For electronic structure calculations with the TB-LMTO-ASA package, see: Andersen *et al.* (1986 ▶).

## Experimental

### 

#### Crystal data

LaZn_12.37_
*M* *_r_* = 947.00
Cubic, 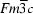

*a* = 12.0940 (9) Å
*V* = 1768.9 (2) Å^3^
*Z* = 8
Mo *K*α radiation
μ = 37.49 mm^−1^
*T* = 293 K
0.05 × 0.03 × 0.01 mm

#### Data collection

Agilent Gemini Ultra diffractometer with Eos CCD detector
Absorption correction: multi-scan (*CrysAlis PRO*; Agilent, 2011 ▶) *T*_min_ = 0.368, *T*_max_ = 1.0
1543 measured reflections
110 independent reflections
108 reflections with *I* > 2σ(*I*)
*R*_int_ = 0.122

#### Refinement

*R*[*F* ^2^ > 2σ(*F* ^2^)] = 0.024
*wR*(*F* ^2^) = 0.052
*S* = 1.22
110 reflections
12 parameters
Δρ_max_ = 0.81 e Å^−3^
Δρ_min_ = −1.08 e Å^−3^

### 

Data collection: *CrysAlis PRO* (Agilent, 2011 ▶); cell refinement: *CrysAlis PRO*; data reduction: *CrysAlis PRO*; program(s) used to solve structure: *SHELXS97* (Sheldrick, 2008 ▶); program(s) used to refine structure: *SHELXL97* (Sheldrick, 2008 ▶); molecular graphics: *DIAMOND* (Brandenburg, 2006 ▶) and *VESTA* (Momma & Izumi, 2008 ▶); software used to prepare material for publication: *publCIF* (Westrip, 2010 ▶).

## Supplementary Material

Crystal structure: contains datablock(s) I, New_Global_Publ_Block. DOI: 10.1107/S1600536811028893/hb5947sup1.cif

Structure factors: contains datablock(s) I. DOI: 10.1107/S1600536811028893/hb5947Isup2.hkl

Additional supplementary materials:  crystallographic information; 3D view; checkCIF report

## Figures and Tables

**Fig. 1.**
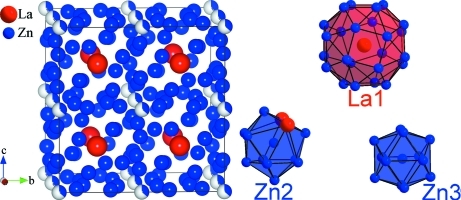
Unit cell projection and coordination polyhedra of atoms in the LaZn12.37 (1) compound

**Fig. 2.**
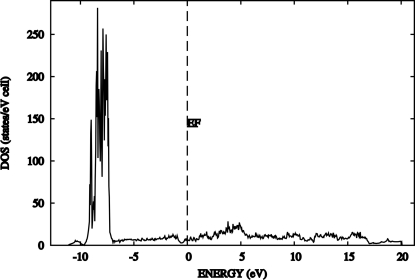
Density of states plot in the LaZn12.37 (1) compound

**Fig. 3.**
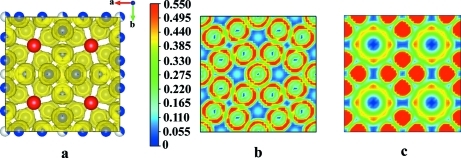
The results of electromic localization function calculations. a - unit cell slice with x=0–1, y=0–1, z=0–0.25 and isosurface drawn at the ELF level=0.5; b - ELF map drawn at z=0; c - ELF map drawn at z=0.25.

## supplementary crystallographic information

### Comment

The results presented in this paper are the part of systematic investigation of ternary rare earth–Zn–Sn systems (see Pavlyuk *et al.*, (2009) and Oshchapovsky *et al.*, (2010)). The corresponding binary La—Zn system is not completely explored yet (Berche *et al.*, 2009).

LaZn_12.37 (1)_ is the only nonstoichiometric compound in the binary La—Zn system. It was found by Rolla *et al.*(Rolla & Iandelli,1941) for the first time. Later Kuz'ma *et al.*, (1966), Veleckis *et al.*,(1967) and Iandelli & Palenzona, (1967) determined the cell parameters of LaZn_12.37 (1)_. These parameters vary a little so Veleckis *et al.* supposed that LaZn_12.37 (1)_ was nonstoichiometric. However, according to Berche *et al. (*2009) the homogeneity range was not determined accurately. Until now there were no single-crystal data indicating positions with partial occupation. In this article we will try to fill this gap. Unit cell projection of the LaZn_12.37 (1)_ compound together with coordination polyhedra of atoms are given in Figure 1. La1 atoms are surrounded by 24 fully occupied positions of zinc atoms forming pseudo-Frank-Kasper polyhedra [LaZn_24_] (CN = 24). Coordination polyhedron of the Zn2 atom is distorted icosahedron [ZnLa_2_Zn_10_] (CN = 12) made of two lanthanum atoms and ten or nine zinc atoms (one position is partially occupied). Zn3 atom in partially occupied position is surrounded by twelve zinc atoms forming isosahedron [ZnZn_12_].

Electronic structure of LaZn_12.37 (1)_ was calculated using TB-LMTO-ASA (Andersen *et al.*, 1986) program package. According to the results of calculations by TB-LMTO-ASA package this compound has metallic bonding (see Fig.2). In this compound the formation of bonds is close to those in Zintl phases, however they have different coordination polyhedra. Lanthanum atoms donate their electrons to zinc atoms. So positive charge density can be observed around lanthanum atoms and negative charge density is around zinc atoms. This indicates that besides of metallic bonding which is dominate in this compound the weak covalent interaction also exists. ELF which indicates bond formation is mostly located at zinc atoms (see Fig. 3 a, b, c). Thus zinc - zinc bonding is much stronger than lanthanum - zinc bonding. So this compound can be treated as insertion of lanthanum atoms into framework made of zinc atoms.

### Experimental

Small irregularly shaped single-crystal of the LaZn_12.37 (1)_ binary compound was selected by mechanical fragmentation of sample with nominal composition LaZn_20_Sn_2._ Alloy was prepared by mixing stoichiometric amounts of powders of zinc, tin and LaZn ligature with subsequent pressing them into pellets. These pellets were enclosed in evacuated silica ampoules and heated in the resistance oven. After that alloys were annealed at 600°C for 30 days and quenched in cold water. No reaction between alloys and quartz containers was observed.

### Refinement

(type here to add refinement details)

### Crystal data

**Table:**

LaZn_12.37_	*D*_x_ = 7.113 Mg m^−^^3^
*M**_r_* = 947.00	Mo *K*α radiation, λ = 0.71073 Å
Cubic, *F**m*3*c*	Cell parameters from 811 reflections
Hall symbol: -F 4c 2 3	θ = 3.4–28.9°
*a* = 12.0940 (9) Å	µ = 37.49 mm^−^^1^
*V* = 1768.9 (2) Å^3^	*T* = 293 K
*Z* = 8	Irregular platelet, grey
*F*(000) = 3426.0	0.05 × 0.03 × 0.01 mm

### Data collection

**Table:**

Agilent Gemini Ultra diffractometer with Eos CCD detector	110 independent reflections
Radiation source: Enhance (Mo) X-ray Source	108 reflections with *I* > 2σ(*I*)
graphite	*R*_int_ = 0.122
ω scans	θ_max_ = 28.3°, θ_min_ = 3.4°
Absorption correction: multi-scan *CrysAlis PRO* (Agilent, 2011)	*h* = −14→16
*T*_min_ = 0.368, *T*_max_ = 1.0	*k* = −16→16
1543 measured reflections	*l* = −9→16

### Refinement

**Table:**

Refinement on *F*^2^	Primary atom site location: structure-invariant direct methods
Least-squares matrix: full	Secondary atom site location: difference Fourier map
*R*[*F*^2^ > 2σ(*F*^2^)] = 0.024	*w* = 1/[σ^2^(*F*_o_^2^) + (0.0097*P*)^2^] where *P* = (*F*_o_^2^ + 2*F*_c_^2^)/3
*wR*(*F*^2^) = 0.052	(Δ/σ)_max_ < 0.001
*S* = 1.22	Δρ_max_ = 0.81 e Å^−^^3^
110 reflections	Δρ_min_ = −1.08 e Å^−^^3^
12 parameters	Extinction correction: *SHELXL97* (Sheldrick, 2008), Fc^*^=kFc[1+0.001xFc^2^λ^3^/sin(2θ)]^-1/4^
0 restraints	Extinction coefficient: 0.00058 (8)

### Special details

**Table:**

Geometry. All e.s.d.'s (except the e.s.d. in the dihedral angle between two l.s. planes) are estimated using the full covariance matrix. The cell e.s.d.'s are taken into account individually in the estimation of e.s.d.'s in distances, angles and torsion angles; correlations between e.s.d.'s in cell parameters are only used when they are defined by crystal symmetry. An approximate (isotropic) treatment of cell e.s.d.'s is used for estimating e.s.d.'s involving l.s. planes.
Refinement. Refinement of *F*^2^ against ALL reflections. The weighted *R*-factor *wR* and goodness of fit *S* are based on *F*^2^, conventional *R*-factors *R* are based on *F*, with *F* set to zero for negative *F*^2^. The threshold expression of *F*^2^ > σ(*F*^2^) is used only for calculating *R*-factors(gt) *etc*. and is not relevant to the choice of reflections for refinement. *R*-factors based on *F*^2^ are statistically about twice as large as those based on *F*, and *R*- factors based on ALL data will be even larger.

### Fractional atomic coordinates and isotropic or equivalent isotropic displacement parameters (Å^2^)

**Table:**

	*x*	*y*	*z*	*U*_iso_*/*U*_eq_	Occ. (<1)
La1	0.2500	0.2500	0.2500	0.0081 (5)	
Zn2	0.0000	0.17786 (6)	0.11938 (6)	0.0113 (4)	
Zn3	0.0000	0.0000	0.0000	0.006 (2)	0.372 (11)

### Atomic displacement parameters (Å^2^)

**Table:**

	*U*^11^	*U*^22^	*U*^33^	*U*^12^	*U*^13^	*U*^23^
La1	0.0081 (5)	0.0081 (5)	0.0081 (5)	0.000	0.000	0.000
Zn2	0.0120 (5)	0.0097 (5)	0.0123 (5)	0.000	0.000	0.0031 (3)
Zn3	0.006 (2)	0.006 (2)	0.006 (2)	0.000	0.000	0.000

### Geometric parameters (Å, °)

**Table:**

La1—Zn2^i^	3.5211 (4)	Zn2—Zn2^ii^	2.6854 (8)
La1—Zn2^ii^	3.5211 (4)	Zn2—Zn2^i^	2.6854 (8)
La1—Zn2	3.5211 (4)	Zn2—Zn2^ix^	2.6859 (12)
La1—Zn2^iii^	3.5211 (4)	Zn2—Zn2^xv^	2.6859 (12)
La1—Zn2^iv^	3.5211 (4)	Zn2—Zn2^xvi^	2.8875 (15)
La1—Zn2^v^	3.5211 (4)	Zn2—La1^xvii^	3.5211 (4)
La1—Zn2^vi^	3.5211 (4)	Zn3—Zn2^xviii^	2.5906 (7)
La1—Zn2^vii^	3.5211 (4)	Zn3—Zn2^xix^	2.5906 (7)
La1—Zn2^viii^	3.5211 (4)	Zn3—Zn2^xx^	2.5906 (7)
La1—Zn2^ix^	3.5211 (4)	Zn3—Zn2^i^	2.5906 (7)
La1—Zn2^x^	3.5211 (4)	Zn3—Zn2^ii^	2.5906 (7)
La1—Zn2^xi^	3.5211 (4)	Zn3—Zn2^xiii^	2.5906 (7)
Zn2—Zn2^xii^	2.5522 (10)	Zn3—Zn2^xxi^	2.5906 (7)
Zn2—Zn2^x^	2.5522 (10)	Zn3—Zn2^xxii^	2.5906 (7)
Zn2—Zn3	2.5906 (7)	Zn3—Zn2^xxiii^	2.5906 (7)
Zn2—Zn2^xiii^	2.6854 (8)	Zn3—Zn2^xvi^	2.5906 (7)
Zn2—Zn2^xiv^	2.6854 (8)	Zn3—Zn2^xiv^	2.5906 (7)
			
Zn2^i^—La1—Zn2^ii^	44.832 (17)	Zn2^ii^—Zn2—Zn2^xv^	161.98 (3)
Zn2^i^—La1—Zn2	44.832 (17)	Zn2^i^—Zn2—Zn2^xv^	108.22 (3)
Zn2^ii^—La1—Zn2	44.832 (17)	Zn2^ix^—Zn2—Zn2^xv^	65.03 (3)
Zn2^i^—La1—Zn2^iii^	163.67 (2)	Zn2^xii^—Zn2—Zn2^xvi^	106.09 (2)
Zn2^ii^—La1—Zn2^iii^	128.82 (2)	Zn2^x^—Zn2—Zn2^xvi^	163.91 (2)
Zn2—La1—Zn2^iii^	146.29 (2)	Zn3—Zn2—Zn2^xvi^	56.130 (16)
Zn2^i^—La1—Zn2^iv^	128.82 (2)	Zn2^xiii^—Zn2—Zn2^xvi^	57.477 (19)
Zn2^ii^—La1—Zn2^iv^	146.29 (2)	Zn2^xiv^—Zn2—Zn2^xvi^	105.27 (2)
Zn2—La1—Zn2^iv^	163.67 (2)	Zn2^ii^—Zn2—Zn2^xvi^	105.27 (2)
Zn2^iii^—La1—Zn2^iv^	44.832 (17)	Zn2^i^—Zn2—Zn2^xvi^	57.477 (19)
Zn2^i^—La1—Zn2^v^	146.29 (2)	Zn2^ix^—Zn2—Zn2^xvi^	57.484 (14)
Zn2^ii^—La1—Zn2^v^	163.67 (2)	Zn2^xv^—Zn2—Zn2^xvi^	57.484 (14)
Zn2—La1—Zn2^v^	128.82 (2)	Zn2^xii^—Zn2—La1	68.752 (6)
Zn2^iii^—La1—Zn2^v^	44.832 (17)	Zn2^x^—Zn2—La1	68.752 (6)
Zn2^iv^—La1—Zn2^v^	44.832 (17)	Zn3—Zn2—La1	117.115 (12)
Zn2^i^—La1—Zn2^vi^	119.133 (3)	Zn2^xiii^—Zn2—La1	173.86 (2)
Zn2^ii^—La1—Zn2^vi^	106.477 (13)	Zn2^xiv^—Zn2—La1	122.82 (4)
Zn2—La1—Zn2^vi^	151.31 (2)	Zn2^ii^—Zn2—La1	67.584 (9)
Zn2^iii^—La1—Zn2^vi^	44.84 (2)	Zn2^i^—Zn2—La1	67.584 (9)
Zn2^iv^—La1—Zn2^vi^	42.496 (13)	Zn2^ix^—Zn2—La1	67.580 (12)
Zn2^v^—La1—Zn2^vi^	78.388 (9)	Zn2^xv^—Zn2—La1	122.80 (3)
Zn2^i^—La1—Zn2^vii^	151.31 (2)	Zn2^xvi^—Zn2—La1	116.657 (11)
Zn2^ii^—La1—Zn2^vii^	119.133 (3)	Zn2^xii^—Zn2—La1^xvii^	68.752 (6)
Zn2—La1—Zn2^vii^	106.477 (13)	Zn2^x^—Zn2—La1^xvii^	68.752 (6)
Zn2^iii^—La1—Zn2^vii^	42.496 (13)	Zn3—Zn2—La1^xvii^	117.115 (12)
Zn2^iv^—La1—Zn2^vii^	78.388 (9)	Zn2^xiii^—Zn2—La1^xvii^	67.584 (9)
Zn2^v^—La1—Zn2^vii^	44.84 (2)	Zn2^xiv^—Zn2—La1^xvii^	67.584 (9)
Zn2^vi^—La1—Zn2^vii^	86.486 (17)	Zn2^ii^—Zn2—La1^xvii^	122.82 (4)
Zn2^i^—La1—Zn2^viii^	106.477 (13)	Zn2^i^—Zn2—La1^xvii^	173.86 (2)
Zn2^ii^—La1—Zn2^viii^	151.31 (2)	Zn2^ix^—Zn2—La1^xvii^	122.80 (3)
Zn2—La1—Zn2^viii^	119.133 (3)	Zn2^xv^—Zn2—La1^xvii^	67.580 (12)
Zn2^iii^—La1—Zn2^viii^	78.388 (9)	Zn2^xvi^—Zn2—La1^xvii^	116.657 (11)
Zn2^iv^—La1—Zn2^viii^	44.84 (2)	La1—Zn2—La1^xvii^	118.34 (2)
Zn2^v^—La1—Zn2^viii^	42.496 (13)	Zn2^xviii^—Zn3—Zn2^xix^	62.436 (7)
Zn2^vi^—La1—Zn2^viii^	86.486 (17)	Zn2^xviii^—Zn3—Zn2^xx^	62.436 (7)
Zn2^vii^—La1—Zn2^viii^	86.486 (17)	Zn2^xix^—Zn3—Zn2^xx^	62.436 (7)
Zn2^i^—La1—Zn2^ix^	42.496 (13)	Zn2^xviii^—Zn3—Zn2	117.564 (7)
Zn2^ii^—La1—Zn2^ix^	78.388 (9)	Zn2^xix^—Zn3—Zn2	117.564 (7)
Zn2—La1—Zn2^ix^	44.84 (2)	Zn2^xx^—Zn3—Zn2	180.00 (3)
Zn2^iii^—La1—Zn2^ix^	151.31 (2)	Zn2^xviii^—Zn3—Zn2^i^	180.00 (3)
Zn2^iv^—La1—Zn2^ix^	119.133 (3)	Zn2^xix^—Zn3—Zn2^i^	117.564 (7)
Zn2^v^—La1—Zn2^ix^	106.477 (13)	Zn2^xx^—Zn3—Zn2^i^	117.564 (7)
Zn2^vi^—La1—Zn2^ix^	146.29 (2)	Zn2—Zn3—Zn2^i^	62.436 (7)
Zn2^vii^—La1—Zn2^ix^	121.00 (2)	Zn2^xviii^—Zn3—Zn2^ii^	117.564 (7)
Zn2^viii^—La1—Zn2^ix^	77.04 (2)	Zn2^xix^—Zn3—Zn2^ii^	180.00 (3)
Zn2^i^—La1—Zn2^x^	78.388 (9)	Zn2^xx^—Zn3—Zn2^ii^	117.564 (7)
Zn2^ii^—La1—Zn2^x^	44.84 (2)	Zn2—Zn3—Zn2^ii^	62.436 (7)
Zn2—La1—Zn2^x^	42.496 (13)	Zn2^i^—Zn3—Zn2^ii^	62.436 (7)
Zn2^iii^—La1—Zn2^x^	106.477 (13)	Zn2^xviii^—Zn3—Zn2^xiii^	67.74 (3)
Zn2^iv^—La1—Zn2^x^	151.31 (2)	Zn2^xix^—Zn3—Zn2^xiii^	62.436 (7)
Zn2^v^—La1—Zn2^x^	119.133 (3)	Zn2^xx^—Zn3—Zn2^xiii^	117.564 (7)
Zn2^vi^—La1—Zn2^x^	121.00 (2)	Zn2—Zn3—Zn2^xiii^	62.436 (7)
Zn2^vii^—La1—Zn2^x^	77.04 (2)	Zn2^i^—Zn3—Zn2^xiii^	112.26 (3)
Zn2^viii^—La1—Zn2^x^	146.29 (2)	Zn2^ii^—Zn3—Zn2^xiii^	117.564 (7)
Zn2^ix^—La1—Zn2^x^	86.486 (17)	Zn2^xviii^—Zn3—Zn2^xxi^	117.564 (7)
Zn2^i^—La1—Zn2^xi^	44.84 (2)	Zn2^xix^—Zn3—Zn2^xxi^	67.74 (3)
Zn2^ii^—La1—Zn2^xi^	42.496 (13)	Zn2^xx^—Zn3—Zn2^xxi^	62.436 (7)
Zn2—La1—Zn2^xi^	78.388 (9)	Zn2—Zn3—Zn2^xxi^	117.564 (7)
Zn2^iii^—La1—Zn2^xi^	119.133 (3)	Zn2^i^—Zn3—Zn2^xxi^	62.436 (7)
Zn2^iv^—La1—Zn2^xi^	106.477 (13)	Zn2^ii^—Zn3—Zn2^xxi^	112.26 (3)
Zn2^v^—La1—Zn2^xi^	151.31 (2)	Zn2^xiii^—Zn3—Zn2^xxi^	117.564 (7)
Zn2^vi^—La1—Zn2^xi^	77.04 (2)	Zn2^xviii^—Zn3—Zn2^xxii^	62.436 (7)
Zn2^vii^—La1—Zn2^xi^	146.29 (2)	Zn2^xix^—Zn3—Zn2^xxii^	117.564 (7)
Zn2^viii^—La1—Zn2^xi^	121.00 (2)	Zn2^xx^—Zn3—Zn2^xxii^	67.74 (3)
Zn2^ix^—La1—Zn2^xi^	86.486 (17)	Zn2—Zn3—Zn2^xxii^	112.26 (3)
Zn2^x^—La1—Zn2^xi^	86.486 (17)	Zn2^i^—Zn3—Zn2^xxii^	117.564 (7)
Zn2^xii^—Zn2—Zn2^x^	90.0	Zn2^ii^—Zn3—Zn2^xxii^	62.436 (7)
Zn2^xii^—Zn2—Zn3	162.22 (4)	Zn2^xiii^—Zn3—Zn2^xxii^	117.564 (7)
Zn2^x^—Zn2—Zn3	107.78 (4)	Zn2^xxi^—Zn3—Zn2^xxii^	117.564 (7)
Zn2^xii^—Zn2—Zn2^xiii^	113.70 (3)	Zn2^xviii^—Zn3—Zn2^xxiii^	112.26 (3)
Zn2^x^—Zn2—Zn2^xiii^	116.33 (3)	Zn2^xix^—Zn3—Zn2^xxiii^	117.564 (7)
Zn3—Zn2—Zn2^xiii^	58.782 (3)	Zn2^xx^—Zn3—Zn2^xxiii^	62.436 (7)
Zn2^xii^—Zn2—Zn2^xiv^	134.159 (17)	Zn2—Zn3—Zn2^xxiii^	117.564 (7)
Zn2^x^—Zn2—Zn2^xiv^	61.64 (4)	Zn2^i^—Zn3—Zn2^xxiii^	67.74 (3)
Zn3—Zn2—Zn2^xiv^	58.782 (3)	Zn2^ii^—Zn3—Zn2^xxiii^	62.436 (7)
Zn2^xiii^—Zn2—Zn2^xiv^	60.0	Zn2^xiii^—Zn3—Zn2^xxiii^	180.0
Zn2^xii^—Zn2—Zn2^ii^	134.159 (17)	Zn2^xxi^—Zn3—Zn2^xxiii^	62.436 (7)
Zn2^x^—Zn2—Zn2^ii^	61.64 (4)	Zn2^xxii^—Zn3—Zn2^xxiii^	62.436 (7)
Zn3—Zn2—Zn2^ii^	58.782 (3)	Zn2^xviii^—Zn3—Zn2^xvi^	117.564 (7)
Zn2^xiii^—Zn2—Zn2^ii^	111.18 (2)	Zn2^xix^—Zn3—Zn2^xvi^	62.436 (7)
Zn2^xiv^—Zn2—Zn2^ii^	65.05 (4)	Zn2^xx^—Zn3—Zn2^xvi^	112.26 (3)
Zn2^xii^—Zn2—Zn2^i^	113.70 (3)	Zn2—Zn3—Zn2^xvi^	67.74 (3)
Zn2^x^—Zn2—Zn2^i^	116.33 (3)	Zn2^i^—Zn3—Zn2^xvi^	62.436 (7)
Zn3—Zn2—Zn2^i^	58.782 (3)	Zn2^ii^—Zn3—Zn2^xvi^	117.564 (7)
Zn2^xiii^—Zn2—Zn2^i^	106.450 (14)	Zn2^xiii^—Zn3—Zn2^xvi^	62.436 (7)
Zn2^xiv^—Zn2—Zn2^i^	111.18 (2)	Zn2^xxi^—Zn3—Zn2^xvi^	62.436 (7)
Zn2^ii^—Zn2—Zn2^i^	60.0	Zn2^xxii^—Zn3—Zn2^xvi^	180.0
Zn2^xii^—Zn2—Zn2^ix^	61.62 (4)	Zn2^xxiii^—Zn3—Zn2^xvi^	117.564 (7)
Zn2^x^—Zn2—Zn2^ix^	134.15 (2)	Zn2^xviii^—Zn3—Zn2^xiv^	62.436 (7)
Zn3—Zn2—Zn2^ix^	103.88 (3)	Zn2^xix^—Zn3—Zn2^xiv^	112.26 (3)
Zn2^xiii^—Zn2—Zn2^ix^	108.22 (3)	Zn2^xx^—Zn3—Zn2^xiv^	117.564 (7)
Zn2^xiv^—Zn2—Zn2^ix^	161.98 (3)	Zn2—Zn3—Zn2^xiv^	62.436 (7)
Zn2^ii^—Zn2—Zn2^ix^	111.90 (3)	Zn2^i^—Zn3—Zn2^xiv^	117.564 (7)
Zn2^i^—Zn2—Zn2^ix^	56.74 (3)	Zn2^ii^—Zn3—Zn2^xiv^	67.74 (3)
Zn2^xii^—Zn2—Zn2^xv^	61.62 (4)	Zn2^xiii^—Zn3—Zn2^xiv^	62.436 (7)
Zn2^x^—Zn2—Zn2^xv^	134.15 (2)	Zn2^xxi^—Zn3—Zn2^xiv^	180.00 (3)
Zn3—Zn2—Zn2^xv^	103.88 (3)	Zn2^xxii^—Zn3—Zn2^xiv^	62.436 (7)
Zn2^xiii^—Zn2—Zn2^xv^	56.74 (3)	Zn2^xxiii^—Zn3—Zn2^xiv^	117.564 (7)
Zn2^xiv^—Zn2—Zn2^xv^	111.90 (3)	Zn2^xvi^—Zn3—Zn2^xiv^	117.564 (7)

Symmetry codes: (i) *y*, *z*, *x*; (ii) *z*, *x*, *y*; (iii) −*z*+1/2, −*y*+1/2, *x*+1/2; (iv) *x*+1/2, −*z*+1/2, −*y*+1/2; (v) −*y*+1/2, −*x*+1/2, −*z*+1/2; (vi) *x*+1/2, *y*, −*z*+1/2; (vii) *y*, −*z*+1/2, *x*+1/2; (viii) −*z*+1/2, *x*+1/2, *y*; (ix) *z*, −*y*+1/2, *x*; (x) *x*, *z*, −*y*+1/2; (xi) −*y*+1/2, −*x*, *z*; (xii) −*x*, −*z*+1/2, *y*; (xiii) −*y*, *z*, −*x*; (xiv) −*z*, *x*, *y*; (xv) −*z*, −*y*+1/2, *x*; (xvi) *x*, *y*, −*z*; (xvii) −*x*, −*y*+1/2, −*z*+1/2; (xviii) −*y*, −*z*, −*x*; (xix) −*z*, −*x*, −*y*; (xx) −*x*, −*y*, −*z*; (xxi) *z*, −*x*, −*y*; (xxii) −*x*, −*y*, *z*; (xxiii) *y*, −*z*, *x*.
